# Comparison between Spatially Resolved Airborne Flux Measurements and Emission Inventories of Volatile Organic Compounds in Los Angeles

**DOI:** 10.1021/acs.est.3c03162

**Published:** 2023-10-04

**Authors:** Eva Y. Pfannerstill, Caleb Arata, Qindan Zhu, Benjamin C. Schulze, Roy Woods, Colin Harkins, Rebecca H. Schwantes, Brian C. McDonald, John H. Seinfeld, Anthony Bucholtz, Ronald C. Cohen, Allen H. Goldstein

**Affiliations:** † Department of Environmental Science, Policy and Management, University of California at Berkeley, Berkeley 94720, California, United States; ‡ Department of Earth and Planetary Science, University of California at Berkeley, Berkeley 94720, California, United States; § Cooperative Institute for Research in Environmental Sciences, University of Colorado Boulder, Boulder 80305, Colorado, United States; ∥ Department of Environmental Science and Engineering, California Institute of Technology, Pasadena 91125, California, United States; ⊥ Department of Meteorology, Naval Postgraduate School, Monterey 93943, California, United States; # NOAA Chemical Sciences Laboratory, Boulder 80305, Colorado, United States; ∇ Department of Chemistry, University of California at Berkeley, Berkeley 94720, California, United States

**Keywords:** air quality, inventory, emissions, fluxes, airborne, volatile organic compounds, California

## Abstract

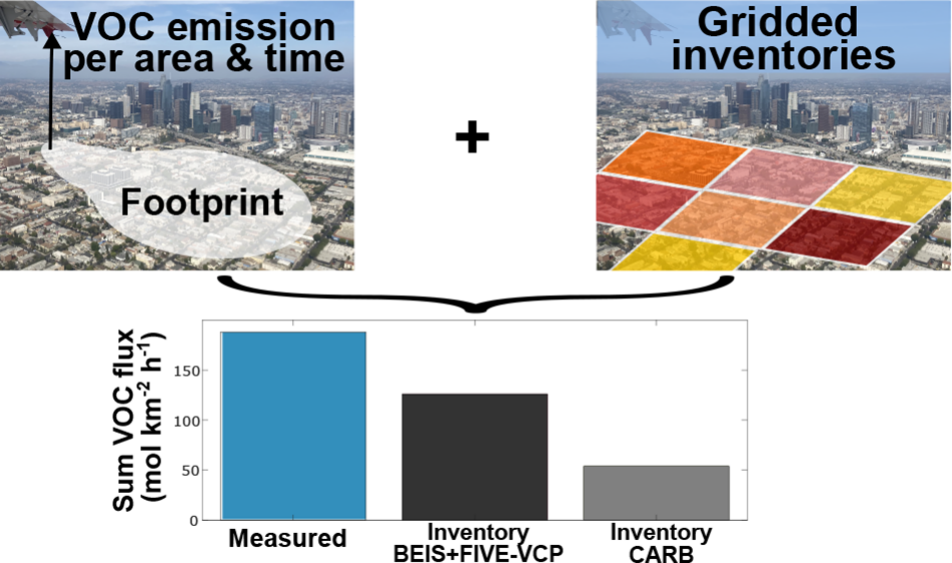

Los Angeles is a major hotspot for ozone and particulate matter air pollution in the United States. Ozone and PM_2.5_ in this region have not improved substantially for the past decade, despite a reduction in vehicular emissions of their precursors, NO_*x*_ and volatile organic compounds (VOCs). This reduction in “traditional” sources has made the current emission mixture of air pollutant precursors more uncertain. To map and quantify emissions of a wide range of VOCs in this urban area, we performed airborne eddy covariance measurements with wavelet analysis. VOC fluxes measured include tracers for source categories, such as traffic, vegetation, and volatile chemical products (VCPs). Mass fluxes were dominated by oxygenated VOCs, with ethanol contributing ∼29% of the total. In terms of OH reactivity and aerosol formation potential, terpenoids contributed more than half. Observed fluxes were compared with two commonly used emission inventories: the California Air Resources Board inventory and the combination of the Biogenic Emission Inventory System with the Fuel-based Inventory of Vehicle Emissions combined with Volatile Chemical Products (FIVE-VCP). The comparison shows mismatches regarding the amount, spatial distribution, and weekend effects of observed VOC emissions with the inventories. The agreement was best for typical transportation related VOCs, while discrepancies were larger for biogenic and VCP-related VOCs.

## Abstract 2

Los Angeles is an air pollution hotspot. Spatially resolved emission measurements of organic gases in Los Angeles show that existing inventories do not accurately predict emissions of some important source categories, with implications for regulatory policies and air quality predictions.

## Introduction

Volatile organic compounds (VOCs), emitted by anthropogenic and biogenic sources, include highly reactive precursors to tropospheric ozone and secondary organic aerosol (SOA). Both ozone and SOA (part of PM_2.5_ fine particulate matter) are air pollutants contributing to respiratory and cardiovascular illnesses. Some VOCs, e.g., benzene, also act as air toxics directly. Air pollution is among the top 10 global health risks,^1^ with 99% of the world’s population breathing air that is deemed unhealthy by the World Health Organization’s standards.^2^ Additionally, tropospheric ozone and SOA have climate effects, with ozone acting locally as a greenhouse gas and SOA contributing to light scattering and cloud formation.

The Los Angeles region frequently exceeds national air quality standards for both ozone and particulate matter.^3^ Emission regulations introduced since the 1970s reduced vehicular VOC emissions,^4^ along with ozone and PM_2.5_ in Los Angeles until ∼2010. Since then, ozone and PM_2.5_ particle pollution have ceased declining.^5,6^ Recent studies suggest that vehicular VOC emissions (while still decreasing) are no longer dominant, and that other VOC sources, such as volatile chemical products (VCPs), are less well understood but becoming more important.^7−9^

Emission inventories are the basis for atmospheric chemistry models used to predict air quality and inform policymaking. The emission data in such inventories usually stem from either bottom-up reporting or are inferred top-down from concentration measurements via chemical transport models. Since these methods do not directly measure regional emissions, they are subject to large uncertainties. Airborne eddy covariance measurements enable the in situ observation of VOC emissions and deposition at spatial resolution of a few km (depending on the flight altitude), which can be used for validation of emission inventories that are at a similar spatial scale.^10,11^ There have been two published studies of airborne VOC emissions over cities, in Mexico City^12^ and London,^11^ and the first VOC airborne eddy covariance study in California was performed in 2011.^13,14^ For airborne eddy covariance measurements, high time resolution (ideally, 10 Hz) of both vertical wind speed and the analyte are necessary. All previously published airborne eddy covariance studies were limited to few VOCs, since the available instrumentation (PTR-quadrupole-MS) was unable to measure several VOCs simultaneously at the necessary time resolution.

In this study, we use the first airborne flux measurements in Los Angeles to map VOC emissions and compare them to two commonly used inventories. State-of-the-art instrumentation allowed for the simultaneous observation of hundreds of VOCs at a 10 Hz time resolution. We observed fluxes of, e.g., oxygenated VOCs, terpenoids, aromatics, siloxanes, and halogenated VOCs. We analyzed the (dis)agreement with the inventories according to relevance for air pollutant formation, amount, spatial distribution, weekend effect, and relationship with population density. Thus, this study provides the first spatially resolved direct evaluation of such a complete suite of VOC emission fluxes in a US urban area.

## Materials and Methods

### Flights and Sampling

Nine flights were conducted over the Los Angeles Basin between June 1–22, 2021, at 300–400 m above ground as part of the RECAP-CA (Reevaluating the Chemistry of Air Pollutants in California) campaign.^15,16^ The 5 h long flights took place between 11:00 and 17:00 local time. Average temperatures ranged from 22 to 30 °C. Figure S1 shows the flight routes and regions covered. Vertical wind speed was measured by a five-hole probe at the nose of the aircraft, and VOC concentrations by a Vocus PTR-ToF-MS (proton transfer reaction time-of-flight mass spectrometer^17^). VOCs were drawn from an ∼6 m long inlet sampling at the nose of the aircraft. The lag time between wind sensor and VOC detection was ∼3 s. Wind and VOCs were recorded at a 10 Hz time resolution. Using airborne eddy covariance with a wavelet analysis approach applying the Morlet wavelet,^12−14,18,19^ the covariance of vertical wind speed and concentrations was converted to spatially resolved VOC fluxes. Details on the aircraft, which has previously been used for airborne eddy covariance measurements,^14,13^ the climatology during the study, the VOC sampling and calibration, the airborne eddy covariance method, and footprint calculations can be found elsewhere.^16,20,21^ Below follows a brief description.

### VOC Measurements and Flux Analysis

The PTR-ToF-MS resolution was ∼4800 with mass spectra recorded from 10 to 500 Da. In-flight zero air measurements were subtracted. Ground calibrations were conducted every 1–3 days using gravimetrically prepared multicomponent VOC standards (Apel-Riemer Environmental Inc., Colorado, USA). The following VOCs were included in the gas standards: methanol, acetonitrile, acetaldehyde, ethanol, acrolein, dimethyl sulfide, isoprene, MACR + MVK, benzene, toluene, xylene, p-cresol, 1-,3-,5-trimethylbenzene, D3 siloxane, D4 siloxane, D5 siloxane, propanol, butanol, acetone, furan, furfural, benzaldehyde, monoterpenes (mixture of α- and β-pinene and limonene), nonanal, acrylonitrile, methyl ethyl ketone, and b-caryophyllene. For all *m*/*z* without a corresponding gas standard, sensitivities were derived from a root function fit to reaction rate normalized sensitivities of nonfragmenting and nonclustering gas-standard calibrated VOCs. The estimated calibration uncertainty for gas-standard VOCs was 20%, and 54% for all other VOCs. Isoprene and acetaldehyde were corrected for interferences from fragmentation of larger molecules,^22^ and benzene was calibrated on *m*/*z* 78.05 to avoid interference of benzaldehyde fragments.^22^ Monoterpenes detected at *m*/*z* 137.13 may include fragments of monoterpenoids with a parent mass of *m*/*z* 155.14 (C_10_H_18_O).^23,24^

Level flight legs of at least 10 km length were chosen for wavelet analysis. Lag times between vertical wind and VOC were determined separately for each VOC and flight segment by searching for the maximum covariance within a 4 s window. Wind and VOC data (10 Hz) were aligned using these lag times. Continuous wavelet transformation, which deconvolutes the covariance within a timeseries throughout both the frequency and time (distance) domains, was applied building up on the work of Karl et al.,^14^ Misztal et al.,^13^ and Wolfe et al.,^25^ and yielded VOC fluxes. For each data point along the flight path, the wavelet transformation of the data produced the local wavelet cospectra. The flux timeseries was created through integration over all frequencies. To remove turbulence-related artificial emission and deposition, a two-sided moving average of 2 km was applied to the 10 Hz fluxes and subsampled to 200 m.

Surface fluxes were calculated from the airborne fluxes by correcting for chemical vertical divergence (i.e., oxidative loss) and physical vertical divergence (loss through horizontal advection and entrainment). The physical vertical divergence was substantial (on average a factor of 2) since there were strong marine winds and a low boundary layer. This led to a relatively large uncertainty contribution to this correction (∼70%). Total uncertainties depend on the VOC, ranging from 75 to 86% for gas-standard calibrated VOCs, and 90–170% for the more than 400 VOCs that were calibrated using the theoretical approach. Average mixing ratios, fluxes, corrections, and uncertainties for each species are listed in Data S1 of Pfannerstill et al.^20^

### Inventory Comparisons

Both inventories used for comparison with the airborne eddy covariance data are at 4 km spatial and hourly time resolution and were computed for the time of the airborne measurements. The California Air Resources Board (CARB) 2021 inventory includes anthropogenic and biogenic, point, and mobile sources. In the CARB inventory, mobile sources are estimated from the EMission FACtor (EMFAC) v1.0.2 and OFFROAD mobile source emission models. The stationary sources are estimated based on a survey of facilities within local jurisdiction and the emission factors from the California Air Toxics Emission Factor database. Biogenic emissions in CARB are from MEGAN 3.0.^26^

The second inventory used here is the sum of the Fuel-based Inventory of Vehicle Emissions combined with Volatile Chemical Products (FIVE-VCP) inventory (anthropogenic) and the Biogenic Emission Inventory System (BEIS) inventory (biogenic), hereafter “BEIS+FIVE-VCP”. We updated BEIS v3.14 for isoprene and monoterpenes from the urban land cover type based on Scott and Benjamin^27^ as done in previous modeling work over Los Angeles.^28^ The fuel-based inventory for vehicle emissions (FIVE), developed by McDonald et al.^29^ and updated by Harkins et al.,^30^ was further revised as described in Coggon et al.^8^ We respeciated the FIVE-VCP inventory to the RACM2_Berkeley2.0 mechanism as described in Pfannerstill et al.^20^ For an expanded description of FIVE-VCP see Text S1. BEIS and FIVE-VCP hourly emissions at 4 km resolution were obtained from the Weather Research and Forecasting model coupled with Chemistry (WRF-Chem v 4.2.2) set up as described in Li et al.^31^ Lumped aromatics that are usually added to benzene, toluene, and xylene in FIVE-VCP were removed for the inventory comparison.

Flux footprints (Figure S1) were calculated using the KL04+ model.^32,33,16^ For comparison with inventories, each footprint (corresponding to a measured flux) was matched to the inventory grid cells that it overlapped with, if the overlap was >10% of the area of the grid cell and the sum of all overlaps amounted to at least 100%. The observations were weighted by overlap and matched by hour and date with the inventory data. For plotting maps, an average of all flyovers was determined for each grid cell.

## Results and Discussion

**Figure 1:**
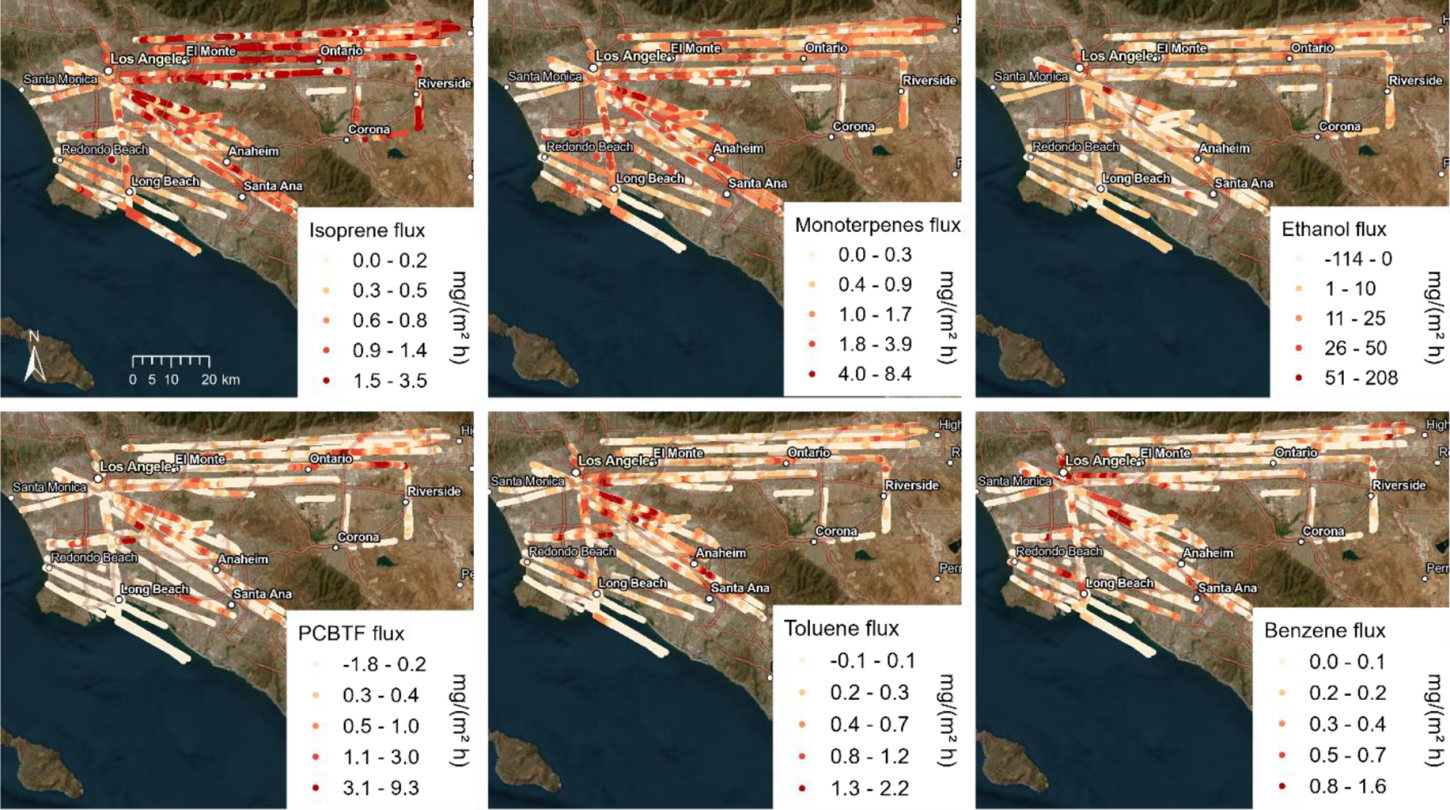
Maps showing fluxes for six examples of VOCs along the flight track. Data from all nine flights are shown here. Values are 2 km running averages downsampled to 100 m.

**Figure 2:**
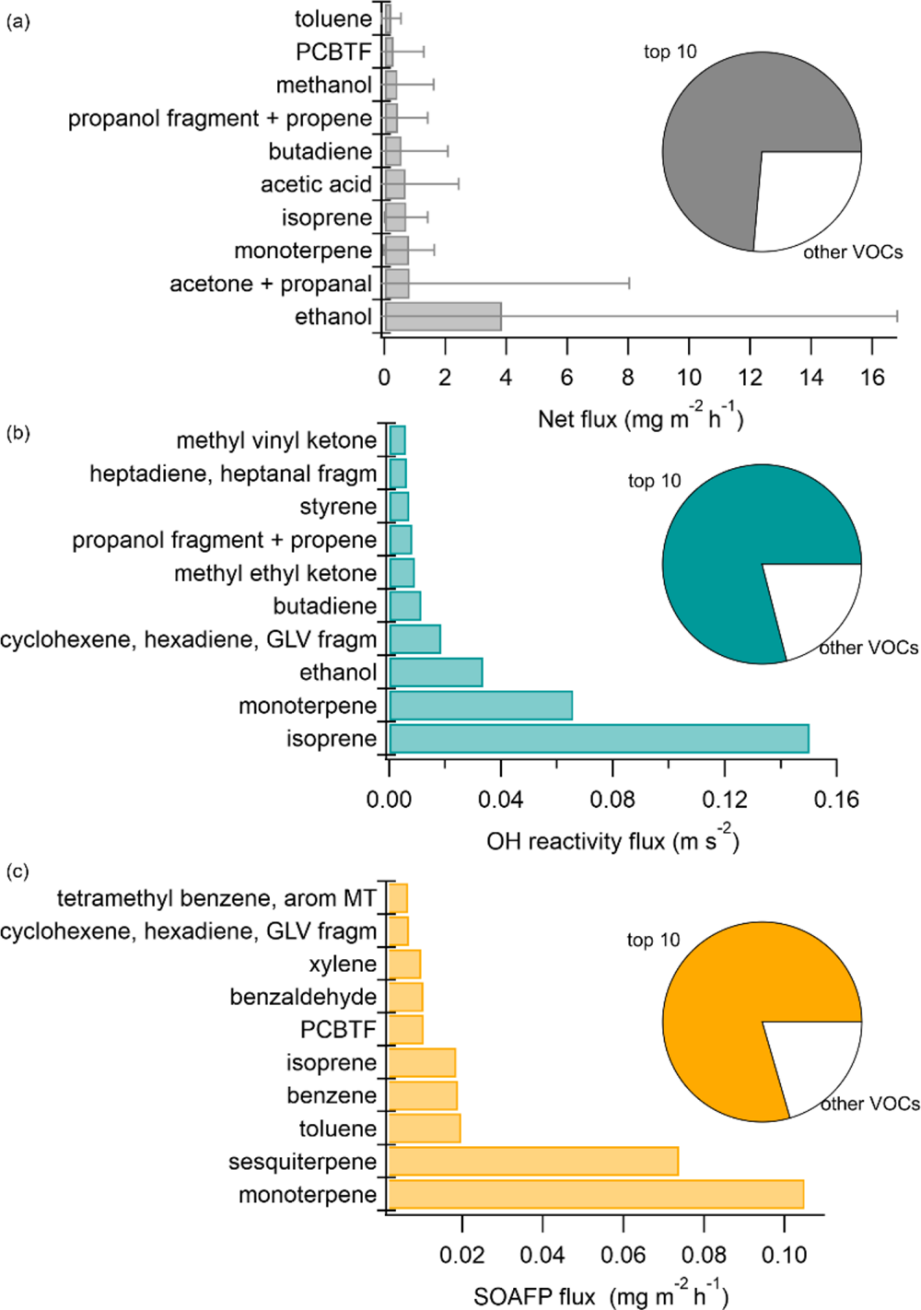
Top 10 measured ions (labeled as attributed VOCs) contributing to the VOC mass flux, OH reactivity flux, and SOA formation potential of flux. arom MT: aromatic monoterpenes, fragm: fragment, GLV: green leaf volatile, and PCBTF: *p*-chlorobenzotrifluoride. Values shown are the averages of the whole campaign. For the net fluxes, the standard deviations are shown as error bars. The pie charts show how much of the summed respective flux is explained by the top 10 VOCs.

The spatial distribution of VOC fluxes measured along flight tracks is shown in Figures 1 and S1 for example species that were highly relevant for mass flux, OH reactivity, and/or SOA formation potential (see Figure 2). Spatial distributions of the fluxes differed strongly between VOCs, reflecting the source distribution, e.g., isoprene emissions were highest on the outskirts of the city, on the less- or nonurbanized hillslopes. This distribution and the observed emission range agrees with a high-resolution BVOC emission inventory for the region.^27^ Monoterpene and sesquiterpene fluxes were higher in downtown Los Angeles than in the San Bernardino Valley, potentially reflecting fragrance-related sources^8,34^ and the distribution of terpene-emitting, non-native trees like eucalyptus.^35^ Ethanol and acetone had a few strong point sources and were, as has been shown previously for OVOCs (oxygenated VOCs),^36^ deposited in some areas (negative fluxes). Benzene was especially high over highways, refineries, and chemical factories, while the distribution of toluene was more similar to that of PCBTF (*para*-chlorobenzotrifluoride), potentially reflecting their similar sources in solvent use.^37^ D5 (decamethylcyclopentasiloxane), a personal care product tracer,^38^ showed a distribution most similar to that of ethanol, reflecting that population density contributes the variability of both.

Measured emissions were in the ranges of direct flux observations performed previously in other cities (Figure S2). Note that we compared our observations only with stationary tower urban flux studies. We refrained from comparing to previous urban airborne eddy covariance observations, since currently available published studies did not correct for vertical divergence.

In the domain, the VOC with the highest observed net mass flux was ethanol, with 3.8 ± 12.9 mg m^–2^ h^–1^ (average ± standard deviation; ∼29% of the total mass flux) (Figure 2). The next highest emissions by mass were the sum of acetone and propanal (C_3_H_6_O, 0.8 ± 7.2 mg m^–2^ h^–1^), monoterpenes (C_10_H_16_, 0.8 ± 0.8 mg m^–2^ h^–1^), and isoprene (0.7 ± 0.7 mg m^–2^ h^–1^). The large standard deviations and mean-to-median ratios (Supplementary Table 3) reflect the high spatial variability in emissions that can be close to zero in some areas and substantial in others. Solvent-related VOCs like acetone, PCBTF and methyl ethyl ketone had particularly large mean-to-median ratios, characteristic of burst-like or localized sources (in contrast to, e.g., methanol, which was more consistently emitted throughout the domain). In terms of contribution to OH reactivity, isoprene was the largest single contributor, followed by monoterpenes and ethanol. The SOA formation potential is highest for VOCs with low-volatility oxidation products, which is why monoterpenes and sesquiterpenes were the most important constituents by a wide margin, followed by toluene. Notably, a coating VCP, PCBTF,^37^ which is not traditionally among surveyed VOCs, appeared in the top 10 both for mass flux and SOA formation potential.

In downtown Los Angeles, we observed an average toluene/benzene ratio of 1.7 (weekend) or 2.2 (weekday). The downtown toluene/benzene emission ratios here were thus very similar to those reported from tunnel measurements in California with 1.99 (weekend) and 2.24 (weekday)^39^ and to direct gasoline exhaust measurements with ∼1.5.^40^ This indicates that vehicle emissions dominate toluene and benzene emissions in downtown Los Angeles. When the whole study area (not just downtown) is included in the average, our flux measurements result in a much larger toluene/benzene ratio of 4.1. This is close to the toluene/benzene ratio of 4.2 found in emissions from solvent use,^41^ and higher than the emission ratios derived from VOC concentration measurements in Los Angeles in 2010,^42^ where the toluene/benzene ratio was 2.9. Potentially, this reflects a growing relative influence of nontraffic (solvent) sources for toluene, while the likely almost exclusively traffic-related benzene emissions^40^ have decreased since 2010. This is supported by the differing spatial distributions of toluene and benzene emissions (Figure 1) and by inventory timelines, which predict that solvent VOC emissions (part of the VCPs) have not decreased as strongly as traffic emissions over the last few decades.^6^

**Figure 3:**
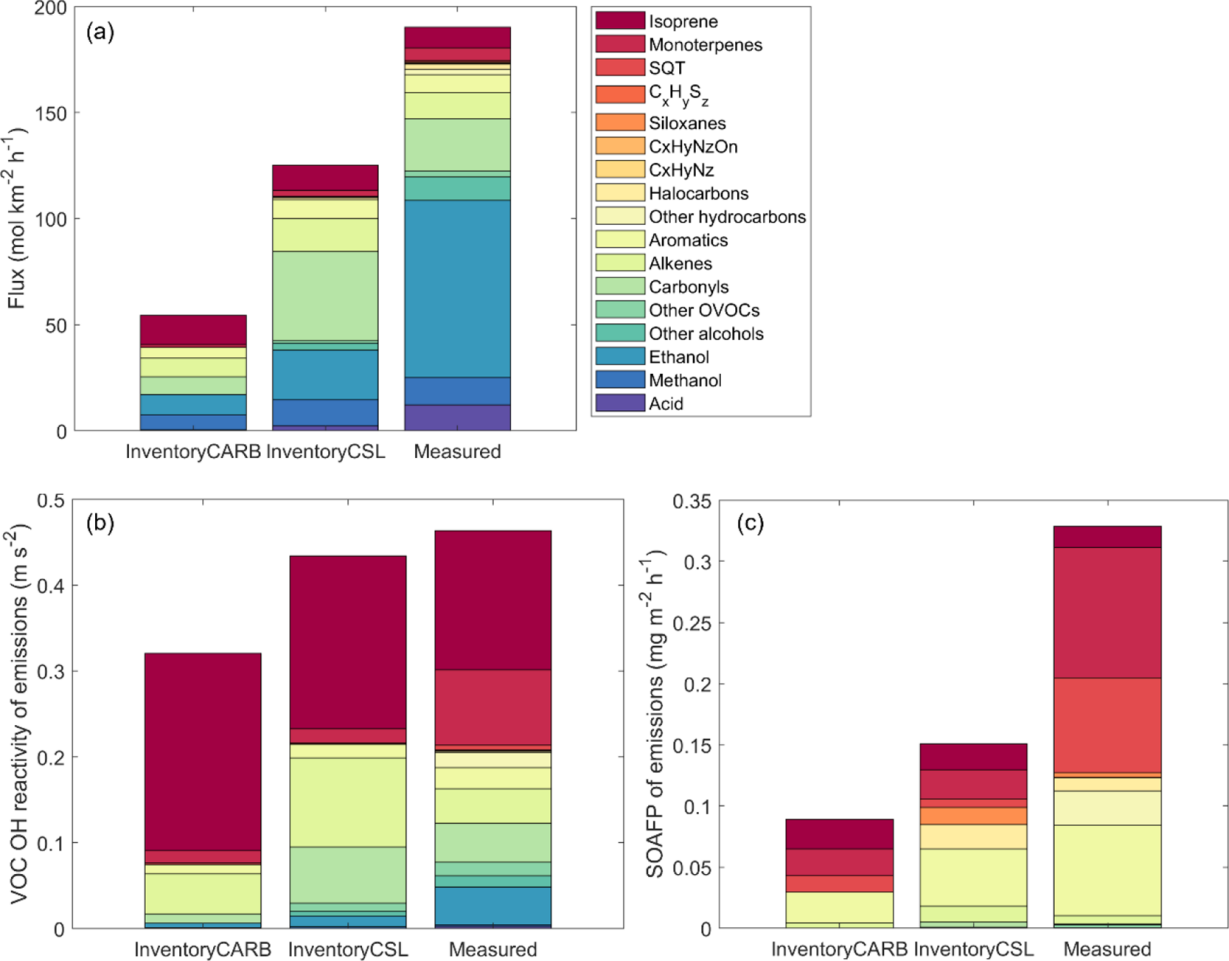
Comparison of campaign average (a) molar flux, (b) VOC OH reactivity, and (c) SOA formation potential of emissions between BEIS+FIVE-VCP (CSL) inventory, CARB inventory, and measurements by chemical family. Alkanes were not included in the comparison since they are difficult to be measured sensitively by PTR-MS.^44^ In both inventories, alkanes contributed ca. 20 mol km^–2^ h^–1^ in emissions, 0.02 ms^–2^ in OH reactivity of emissions, and a negligible amount in the SOA formation potential of emissions. Apart from alkanes, this comparison includes all VOCs available in the observations or in the inventories.

The flux measurements were compared with two different emission inventories: (1) the CARB inventory, which includes biogenic emissions from MEGAN 3.0 and anthropogenic emissions, and (2) the sum of the FIVE-VCP (anthropogenic) and BEIS (biogenic) inventories. For this purpose, the flux footprints were matched spatially with the 4 km × 4 km grid cells of the inventories (see methods) and in time according to the exact same day and hour of the inventory emissions. Figure 3a displays a comparison of the sum of measured molar VOC emissions with those included in the inventory from the averages of the whole campaign. There is a difference of a factor of ∼2 between the inventories in the summed molar flux (54 mol km^–2^ h^–1^ for CARB vs 126 mol km^–2^ h^–1^ for BEIS+FIVE-VCP), and the observed sum (190 mol km^–2^ h^–1^) is ca. 50% higher than the BEIS+FIVE-VCP prediction. Note that the uncertainty of individual VOC fluxes introduced through the necessary vertical divergence correction is on the order of a factor of 2. The observed ethanol emissions (which contributed ∼half of the summed molar VOC flux) were much higher than both inventories predicted—more than a factor of 4 compared to the BEIS+FIVE-VCP inventory and more than a factor of 5 compared to the CARB inventory. The sum of observed acid emissions was also substantially (more than a factor of 5) underestimated by the inventories, suggesting that cooking may be an important source of the mismatch. The summed carbonyl emissions were higher than observations in the BEIS+FIVE-VCP and lower than observations in the CARB inventory. Overall, the total observed oxygenated VOC emissions were substantially underestimated by the inventories. A similar observation was made in the comparison of VOC flux measurements with a regional inventory in London.^43^

In Figure 3b, the same data are shown in terms of OH reactivity, based on OH reaction rate constants listed in Data S1 of Pfannerstill et al.^20^ The OH reactivity emission sums are comparable between inventories and measurements. However, the inventories included more isoprene (within the measurement uncertainty) and substantially less monoterpene and alcohol emissions than the observations, leading to a similar sum based on a composition different than observed. Monoterpenes contributed 19% of the OH reactivity sum in the observations, much higher than in the inventories, where they contributed only 4% (BEIS+FIVE-VCP) or 5% (CARB). The alcohol contribution was 13% in the observations but only 5% (BEIS+FIVE-VCP) or 2% (CARB) in the inventories. The isoprene contribution to OH reactivity fluxes was 35% in the observations but 46% (BEIS+FIVE-VCP) or 72% (CARB) in the inventories. We note that since the PTR-MS method is not able to measure all VOCs (notably it is unable to ionize alkanes), there may be a significant missing OH reactivity source. Based on direct total OH reactivity observations performed in Los Angeles in 2010,^45^ and after comparison with species observed then, we estimate this missing OH reactivity source to be at maximum ∼30% (uncorrected for trends since 2010).

**Figure 4:**
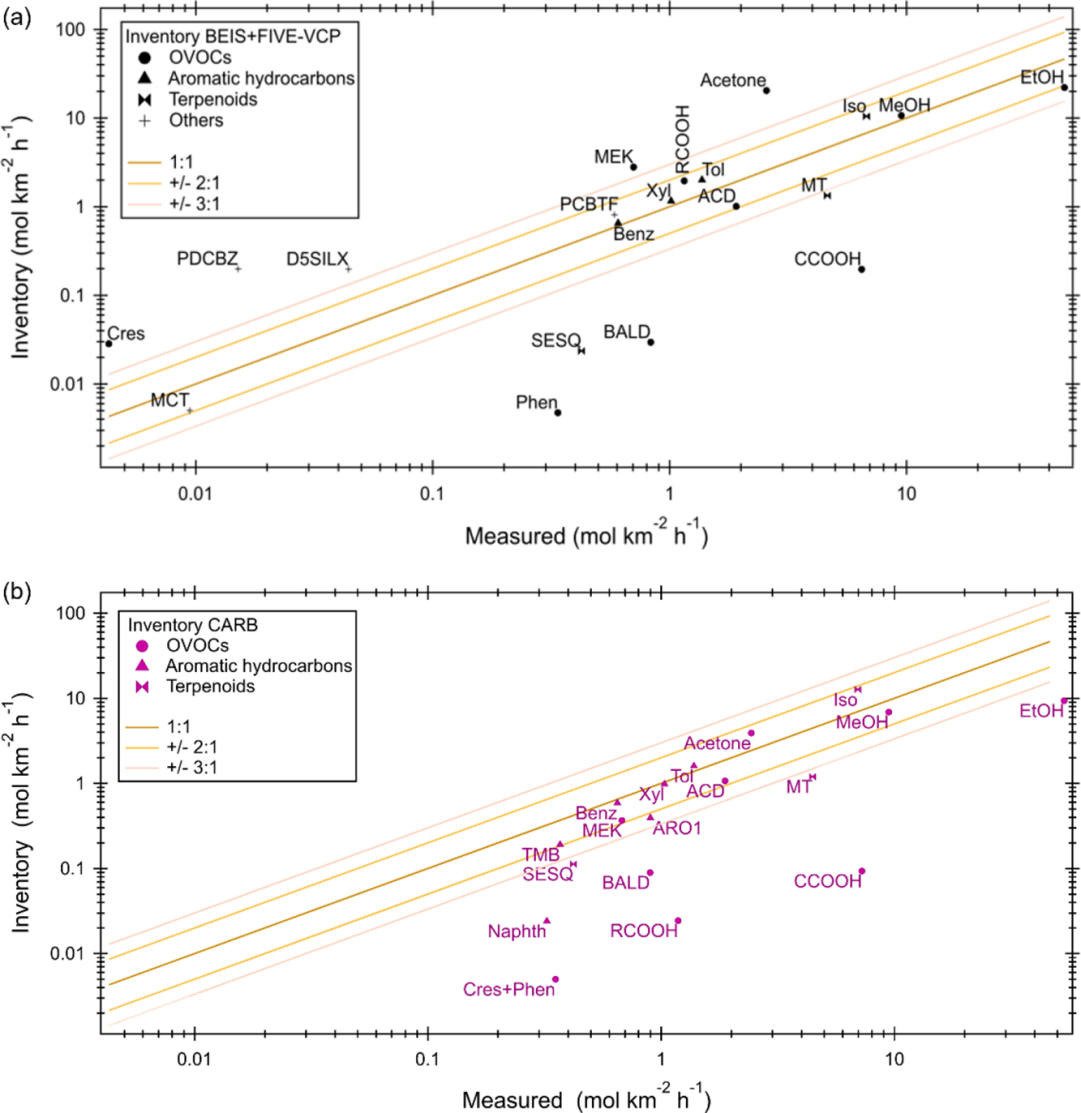
Comparison of median values between measured and inventory emissions of individual VOCs for (a) BEIS+FIVE-VCP and (b) CARB. Cres: cresol, Phen: phenol, MCT: methanethiol, PDCBZ: paradichlorobenzene, D5SILX: D5 siloxane, SESQ: sesquiterpenes, TMB: trimethylbenzene, BALD: benzaldehyde, Naphth: naphathalene, MEK: methyl ethyl ketone, Benz: benzene, PCBTF: para-chlorobenzotrifluoride, Xyl: xylene, Tol: toluene, MT: monoterpenes, Iso: isoprene, MeOH: methanol, EtOH, ethanol, CCOOH: acetic acid, ACD: acetaldehyde, RCOOH: higher organic acids, and ARO1: other aromatics with kOH <2 × 10^4^ ppm^–1^ min^–1^. “Measured” values can slightly differ in comparison to each inventory because of a different distribution and coverage of inventory grid cells (Figure S4: same figure for mean values).

SOA formation potentials of the emitted VOCs were estimated using the statistical oxidation model, which is based on SOA yields from chamber studies and approximately accounts for multigenerational aging,^46^ combined with a one-dimensional volatility basis set for OVOCs.^47^ The overall SOA formation potential of emitted VOCs (Figure 3c) was underestimated substantially (by a factor of 2–3) by both inventories. This discrepancy was mainly due to underestimated mono- and sesquiterpene emissions, which were on average at least a factor of 5 higher than in the inventories. This caused the fractional contribution of monoterpenes to SOA formation potential to be 33% in the observations but just 15% (BEIS+FIVE-VCP) or 24% (CARB) in the inventories. On the other hand, the contribution of aromatic emissions was well represented by the FIVE-VCP inventory. The CARB inventory underestimated the aromatic contribution because, despite a good match for simple aromatics (Figure 4), it underestimated heavier aromatic VOCs (see, e.g., naphthalene in Figure 4). The summed SOA formation potential is likely underestimated by our observations, because long-chain alkanes, as well as highly oxidized intermediate-volatility organic compounds, which were not detected in our measurements, are relevant SOA precursors.^48^ We estimate that including these would increase the total SOA formation potential of emissions by a maximum ∼30%.

A comparison of median fluxes of individual VOC species (Figure 4) provides a more detailed view of the similarities and differences between the two inventories and the measurements. The general trends are the same in comparison to median values (Figure 4) and mean values (Figure S4, Supplementary Table 1). Since the uncertainties of the observed VOC fluxes introduced through the necessary vertical divergence correction are on the order of a factor of 2, the agreement between measurements and inventories was considered reasonable within that range (Figure 4). Note that this uncertainty is likely systematic and a single scale factor for all measurements, so a better knowledge of it would not reduce the spread of agreement. The distribution of points shows that the CARB inventory has a general tendency toward underestimation for a subset of compounds (Figure 4b), while the BEIS+FIVE-VCP inventory scatters more around the 1:1 line (Figure 4a) both in the positive and the negative direction.

The agreement between the medians from measurements and both inventories was excellent for benzene, toluene, and xylene. In addition, within a factor of 2 and thus within the uncertainty were (for both inventories) methanol, isoprene, acetaldehyde, and PCBTF (for BEIS+FIVE-VCP), as well as trimethylbenzene and acetone (for CARB). It must be noted that agreement may occur for the wrong reasons (i.e., overestimation of some sources while underestimating others in the inventory) and that the uncertainty of the measurements implies that even VOCs that match well within the uncertainty here might have additional sources not included in the inventory. The comparison of averages (Figure S4) indicates that localized high emissions of aromatics may be underestimated by the inventories. Larger discrepancies were observed for benzaldehyde, phenol, cresol, ethanol, acids, sesquiterpenes, dichlorobenzene, D5 siloxane, and methanethiol. Among these, benzaldehyde, ethanol, acids, and sesquiterpenes were important contributors to OH reactivity or SOA formation potential (Figure 2). The underestimation of naphthalene by the CARB inventory may be related to its nontraditional sources as a VCP in household pesticides^49^ or from asphalt.^50,51^ Generally, there was better agreement between observations and inventories for aromatics than for OVOCs and other VCPs. This reflects the fact that routine measurements historically have been more focused on typical traffic emissions, such as aromatics, which are therefore better understood.

Conversely, OVOCs were not easily measurable by routine methods in the past. For example, even during an intense, state-of-the-art observation campaign like CalNex 2010 in Los Angeles, only nine OVOC species were observed.^45^ With the airborne flux measurements in 2021, we observed significant fluxes of 93 different OVOCs thanks to more comprehensive state-of-the-art instrumentation. Eight of these OVOCs were among the 10 most important VOCs in terms of mass flux or OH reactivity flux (Figure 2). The CARB inventory includes 19 and the BEIS+FIVE-VCP inventory contains 17 OVOCs (although including some lumped species). In the comparison of OVOCs, it is important to be aware that our observations are net fluxes, which means that the median is decreased due to deposition fluxes. Emission inventories do not assume deposition, which is treated separately by another module in atmospheric modeling. Thus, gross emission fluxes would be higher than the observation medians shown in Figure 4—in some cases (e.g., for acetone or ethanol) substantially, because of large deposition fluxes (Figure 6).

Notably, some relevant VOCs are included in only one of the inventories. For example, PCBTF, a solvent VOC that is included, e.g., in coatings,^37^ is listed as carcinogenic^52^ and contributed a substantial amount to SOA formation potential (Figure 3). However, PCBTF is missing from the CARB inventory and was only recently added to the FIVE-VCP inventory. In addition, missing from the CARB inventory and recently added to FIVE-VCP is dichlorobenzene (“PDCBZ” in Figure 4), which is listed as carcinogenic^52^ and widely used in pesticides.

Figure S3 demonstrates the importance of the discrepancies between observations and inventories of distinct VOC classes for the total mass flux, OH reactivity flux, and SOA formation potential flux. For the mass flux, the difference between observations and inventories was largest in alcohols (mainly ethanol), followed by other oxygenated VOC classes (carbonyls for CARB and acids for BEIS+FIVE-VCP). The largest missing sources of OH reactivity (and thus ozone formation potential) were monoterpenes and alcohols in both inventories. However, since this missing source was made up for by an overestimation of isoprene emissions (CARB) and/or alkene emissions (BEIS+FIVE-VCP), the summed OH reactivity source was very similar between observations and inventories (Figure 3). The largest missing contributors to the SOA formation potential were monoterpenes and sesquiterpenes in both inventories.

**Figure 5:**
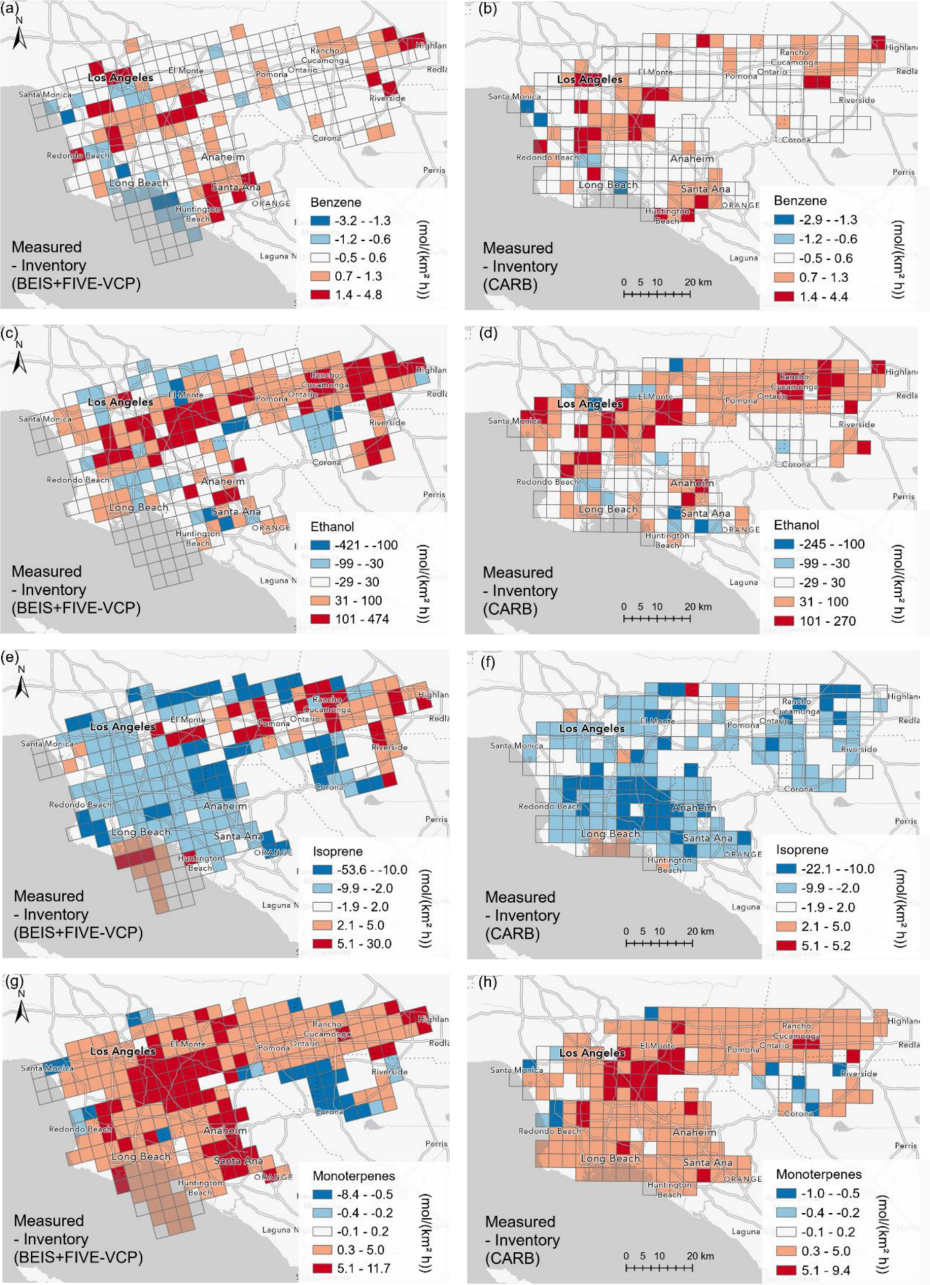
Difference of flux measured-inventory for (a) BEIS+FIVE-VCP, benzene, (b) CARB, benzene, (c) BEIS+FIVE-VCP, ethanol, (d) CARB, ethanol, (e) BEIS+FIVE-VCP, isoprene, (f) CARB, isoprene, (g) BEIS+FIVE-VCP, monoterpenes, and (h) CARB, monoterpenes. Blue colors show that the measurements were lower than the inventory, red colors show that the measurements were higher than the inventory. A comparison by ratio instead of difference for the same VOCs is shown in Figure S5.

While medians of the whole campaign (Figure 4) can provide an overview validation of the inventories, spatially resolved airborne flux observations can be used more specifically to map regional agreements and disagreements with the emission inventories. Figure 5 shows the difference in flux units between inventories and measurements on a 4 × 4 km grid scale for benzene, ethanol, isoprene, and monoterpene fluxes. Both inventories agreed relatively well with the benzene observations in most of the domain, except for some underestimation in the downtown area. For benzene, the differences between both inventories and measurements were small in the eastern San Bernardino Valley (around Rancho Cucamonga). However, in relative terms, this was the region where the inventories, especially CARB, underestimated the benzene emissions most clearly (Figure S5). In the same region, the CARB inventory also underestimated NO_*x*_ fluxes,^15^ suggesting a common source of the mismatch. This may be added truck traffic from “mega warehouses” for the online shopping economy that have been added to the region recently.^53^ For ethanol, the spatial pattern of disagreement with the observations is almost the same between the two inventories. There appears to be a significant missing source of ethanol, which we suspect to be cooking and possibly other indoor-to-outdoor emissions combined with point sources (e.g., breweries and food manufacturing). Isoprene emissions matched best with both inventories in the San Bernardino Valley, while the observations were lower than inventories in parts of the region between downtown and Santa Ana. For monoterpene emissions, there is almost no grid cell with a reasonable match between the observations and inventories. The largest underestimation by the inventories (in absolute terms) occurred in an area that includes much of downtown Los Angeles. In relative terms, both inventories were more than a factor of 4 lower than the measurements also in the San Bernardino Valley and much of the Santa Ana region. Potential explanations for this mismatch are (1) a missing anthropogenic source of monoterpenes from fragrance use, and (2) a (probably larger) missing biogenic source induced by flowering and/or drought stress as well as an unrealistic plant species composition and distribution in the inventories.^20^ We provide more maps of the inventory and measurement values as well as the differences between both online^54^ and tabular regional average comparisons in Supplementary Table 1.

**Figure 6:**
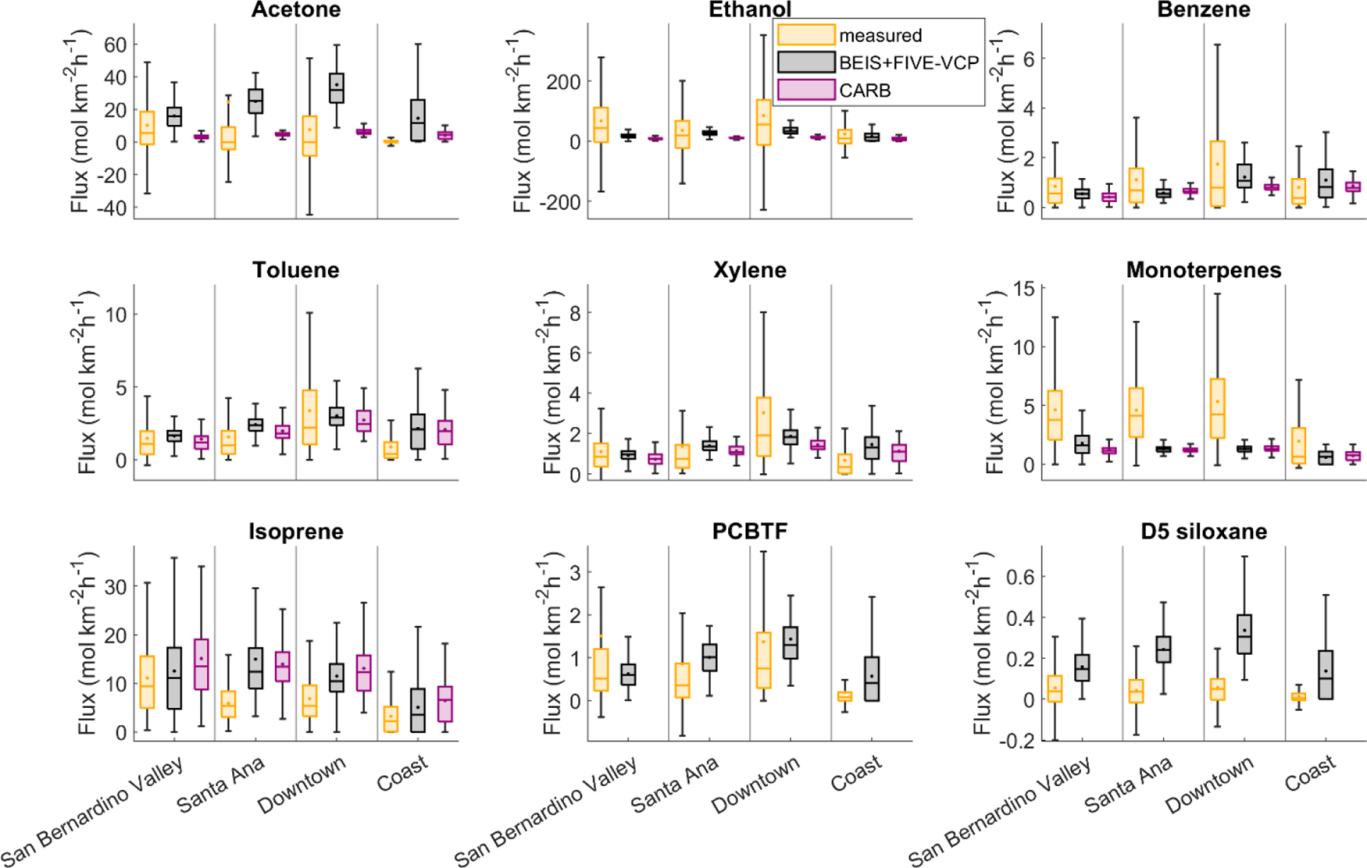
Regional fluxes are shown for a selection of VOCs in comparison between measurements and the two inventories. The boxes represent the 25th–75th percentile of the data, the whiskers represent the 5th–95th percentile, the circles represent the mean, and the horizontal lines represent the median. PCBTF and D5 siloxane emissions are not reported in the CARB inventory.

The fact that observed fluxes matched better with the inventories in some regions than others is also illustrated in Figure 6 and Supplementary Table 1. Observed benzene fluxes agreed with both inventories to within 50% in all regions. However, for example, isoprene fluxes agreed with the BEIS+FIVE-VCP inventory within 10% in the San Bernardino Valley, within 50% in at the coast, but only within a factor of 2 in Downtown and Santa Ana. Similarly, the CARB inventory agreed with the observations within 30% in the San Bernardino Valley, but only within a factor of 2–3 in the other regions. Notably, for many of the VOCs shown, the match was best in the San Bernardino Valley. Potentially, this may be related to the fact that intense VOC observation campaigns in Los Angeles have so far usually been performed in Pasadena,^42,39^ which is located in the San Bernardino Valley. Inventories have been validated against those measurements.^28,42^ Another factor likely contributing to mismatches in polar VOCs is that our observations are net fluxes, while the inventories consider only emissions and not deposition. This means that some VOCs that are strongly deposited (e.g., acetone and other OVOCs, or D5 siloxane, Figure 6) have a reduced net flux as opposed to if only the emissions were considered. In addition to that, for D5 siloxane, it is possible that the measurements somewhat underestimated the fluxes because siloxanes are relatively sticky in the inlet system, leading to a dampened covariance peak. Additionally, our measurements were conducted outside the typical time window when siloxane personal care product emissions are highest (morning^38^).

Some of the mismatch may be due to faulty assumptions of temperature dependence of emissions in the inventories^20^ or due to differences in temperature assumed in the inventories.

In order to investigate the effect of working days versus weekends on emissions, we performed three out of nine flights on weekends. Figure S6 shows comparisons between weekend and weekday fluxes for the measurements and between the two inventories for a selection of VOCs. A tabular overview of weekend–weekday comparisons is given in Supplementary Table 2. Generally, there is a large overlap of the interquartile ranges between weekdays and weekends. Benzene, which mainly is emitted from vehicles, did not display any significant weekend effect. This observation agrees with the inventories and with previous studies that demonstrated no significant difference in gasoline-fueled vehicle activity between weekdays and weekends in California.^55,56^ Isoprene, which as a biogenic emission is expected to be independent of workdays, also exhibits no weekend effect.

However, the median and top 50% fluxes of a number of other VOCs decreased on the weekend. This includes several of the more VCP- or solvent-related VOCs: acetone (94% decrease in median), ethanol (44% decrease), toluene (23% decrease), xylenes (26% decrease), and PCBTF (62% decrease). Since much solvent use is work-related (e.g., construction, printing, and cleaning), this observed decrease in emissions on weekends seems plausible. It is also notable that the decreases in the aromatics xylene and toluene were smaller than in the purely solvent-sourced PCBTF, since part of the aromatic emissions are expected to come from gasoline vehicles and therefore not be affected as much by the weekend (see benzene). The inventories do not appear to consider a weekend effect on solvent emissions, with insignificant weekday–weekend differences (below 9%) in the medians for the VOCs listed above. This impacts the OH reactivity and SOA projections.

Figure S7 explores relationships between observed VOC emissions and population density for VOCs whose indoor-to-outdoor emission fraction is large (Arata et al., in preparation): ethanol, D5 siloxane, acetic acid, and acetaldehyde. These VOCs are expected to have other sources besides residences, e.g., acetaldehyde also comes from fossil fuel combustion^57^ or solvent use,^41^ and ethanol is also expected to be released from biogenic sources,^58^ fuel combustion or evaporation,^59^ solvents,^60^ restaurants and food/alcohol manufacturing point sources. Moreover, any population density effects are superimposed with weekend or temperature effects. This makes it reasonable that the emission relationship with the population density shows a large scatter. Due to the large scatter of the raw data, the tendency of increased emissions of these VOCs with population density is statistically not significant. However, the median emissions of these VOCs increase within the 1σ uncertainty with binned population density (Supplementary Table 4). This result agrees with a study that found a population density dependence of VOC mixing ratios for several VCPs, including D5 siloxane.^60^

The BEIS+FIVE-VCP inventory shows a larger relative increase in ethanol and D5 siloxane emissions with population density than the observations but a smaller relative increase in acetaldehyde emissions and none in acetic acid emissions. The CARB inventory reflects the observed relative ethanol emission increase well, while its increases in acetic acid and acetaldehyde emissions are somewhat smaller. Overall, the relative dependence of these VOC emissions on population density is mostly well reflected in the inventories. The absolute amounts emitted are a more important mismatch (see Figure 4).

In conclusion, the overall underestimation of alcohol, monoterpene, and sesquiterpene emissions by both inventories that we validated (CARB and BEIS+FIVE-VCP) is relevant for air quality predictions, since alcohols and monoterpenes contributed 13 and 19%, respectively, to OH reactivity (relevant for ozone formation), and sesquiterpenes and monoterpenes contributed 23 and 32%, respectively, to SOA formation potential. Our measurements indicate important missing sources of ethanol and terpenoids that should be added to current inventories. Temperature effects in anthropogenic VOC emissions not incorporated in the inventories play a role in the mismatches.^20^ Traditionally better quantified typical traffic emissions such as aromatics matched better between the inventories and measurements than did OVOCs and terpenoids. Generally, the CARB inventory had a tendency toward underestimation of VOC emissions, while the BEIS+FIVE-VCP inventory underestimated some and overestimated other VOCs.

Apart from these general trends, there were regional trends in the mismatches. For many VOCs, the inventories agreed best with the observations in the inland region of the San Bernardino Valley, while downtown Los Angeles was more prone to emission underestimations, e.g., for ethanol and monoterpenes.

Our results point to the necessity to improve inventory emissions of nontraditional VOC sources like VCPs, solvent use, and cooking to obtain a comprehensive representation of relevant air pollutant precursors in urban areas. They also show the need for a better representation of urban biogenic VOC emissions in inventories since these are highly important for ozone and SOA formation and not as accurately represented as transportation emissions.

## Supporting Information Available

Zonal statistics of VOC emissions from observations and inventories (XLSX)
Weekday/weekend statistics of VOC emissions from observations and inventories (XLSX)
Additional details on the model, and supplementary figures and tables with analyses as referenced in the text (PDF)
