# Supplementary material for: Comparison between Spatially Resolved Airborne Flux Measurements and Emission Inventories of Volatile Organic Compounds in Los Angeles
Source: Environ Sci Technol. 2023 Oct 4;57(41):15533–45. doi: 10.1021/acs.est.3c03162 (PMC10586371; doi:10.1021/acs.est.3c03162)
Supplement: Supplementary file 3 — es3c03162_si_003.pdf [file es3c03162_si_003.pdf]

Supplementary Information for

Comparison between spatially resolved airborne flux measurements and emission inventories of volatile organic compounds in Los Angeles

Eva Y. Pfannerstill[1,], Caleb Arata[1], Qindan Zhu[2,3,†], Benjamin C. Schulze[4], Roy Woods[5], Colin Harkins[3,6], Rebecca H. Schwantes[6], Brian C. McDonald[6], John H. Seinfeld[4], Anthony Bucholtz[5], Ronald C. Cohen[2], Allen H. Goldstein[1,*]*

[1] Department of Environmental Science, Policy and Management, University of California at Berkeley, Berkeley 94720, California, USA

[2] Department of Chemistry, University of California at Berkeley, Berkeley 94720, California, USA

[3] Cooperative Institute for Research in Environmental Sciences, University of Colorado Boulder and NOAA Chemical Sciences Laboratory, Boulder 80305, Colorado, USA

[4] Department of Environmental Science and Engineering, California Institute of Technology, Pasadena 91125, California, USA

[5] Department of Meteorology, Naval Postgraduate School, Monterey 93943, California, USA

[6] NOAA Chemical Sciences Laboratory, Boulder 80305, Colorado, USA

Contents:

Text S1: Description of FIVE-VCP Inventory p. S2

Figures S1-S8 p. S3 - S10

Supplementary Table 3 p. S11

Supplementary Table 4 p. S12

References p. S13

Separate Supplementary Files:

Supplementary Table 1 (xlsx)

Supplementary Table 2 (xlsx)

Text S1: Description of FIVE-VCP Inventory

Mobile source emissions (on-road and off-road engines using gasoline or diesel) are taken from the Fuel-based Inventory of Vehicle Emissions or FIVE (*1, 2*). Briefly, gasoline and diesel fuel sales for on-road and off-road engines are reported by state each year. Monthly regional fuel sales and monthly state-level traffic estimates are used to update this with monthly adjustments to fuel sales at a state-level for on-road and a regional level for off-road (*3*). For on-road engines, fuel sales are downscaled to the roadway level using light and heavy-duty vehicle count data where possible (~70% of gasoline and ~80% of diesel nationally) with the remaining portion of fuel sales being distributed spatially using population density and the result mapped onto a 4x4 km grid for the contiguous United States (McDonald et al., 2014). With the mapped fuel, co-emitted air pollutant emissions are estimated using fuel-based emission factors (e.g, g/kg fuel) for gasoline and diesel engines from roadside measurements (*4, 5, 2*). Day-of-week and diurnal adjustments are made separately for light-duty gasoline and heavy-duty diesel vehicles to estimate hourly emissions (*1*). Similarly, off-road fuel sales are spatially and temporally (with hourly, day-of-week and seasonal adjustments incorporated) allocated according to the NEI 2017 (*6*). Fuel-based emission factors are again used to calculate hourly emissions (*2*).

Volatile Chemical Product (VCP) emissions are estimated following McDonald et al. (*7*), where a mass balance of the petrochemical chemical industry was performed to estimate per capita use of adhesives, coatings, cleaning agents, inks, pesticides and personal care products. Emission factors are applied to these categories, as described in Coggon et al. (2021), to produce a 4x4km gridded inventory for the US. Oil and gas emissions are taken from the Fuel-based Oil and Gas Inventory (FOG, (*8*)). Other area and point source emissions make up only a small fraction of anthropogenic VOC emissions (*9*) and are taken from the NEI 2017 (*6*). Emissions from the NEI

2017 are adjusted monthly using relevant economic and energy datasets for the U.S. (He et al., in preparation). The combination of these inventories is referred to here as the as the FIVE-VCP inventory.

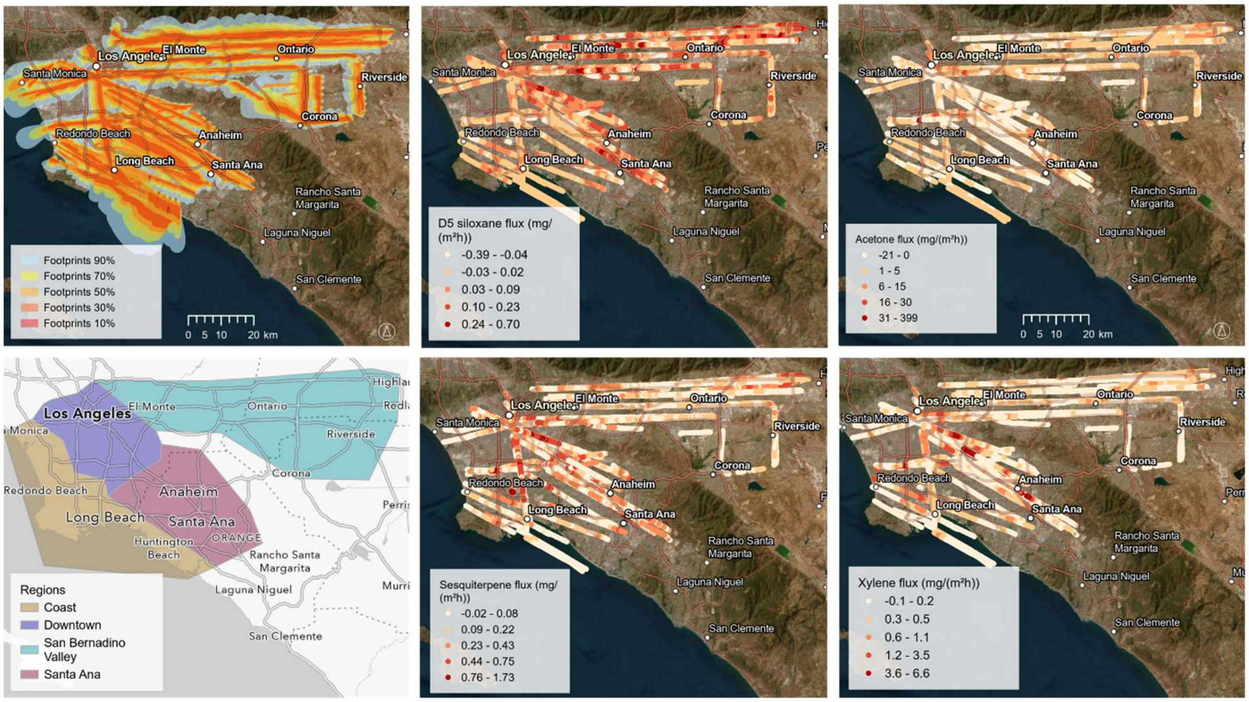

Figure S1. Maps showing flux footprints, regions defined for the analysis, and fluxes for four example VOCs along the flight track.

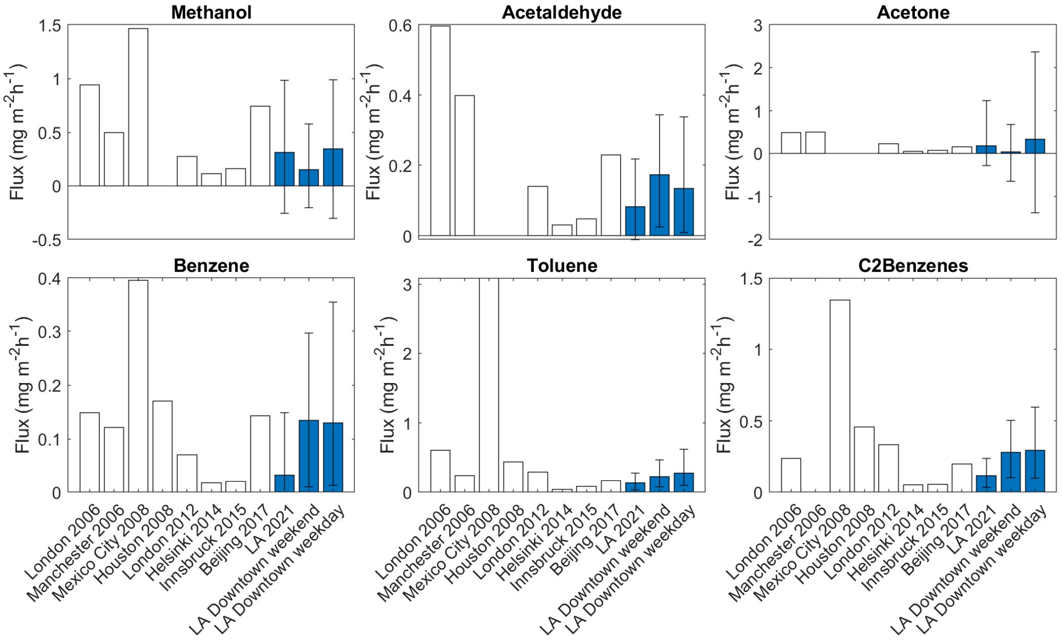

Figure S2. Comparison of median flux observations from this airborne study (blue) with previous stationary tower urban flux observations. Error bars show the 25th to 75th percentiles. C2-benzenes include xylenes and ethylbenzene. References: *10–17*. Note that the Houston study measured fluxes using relaxed eddy accumulation, not eddy covariance.

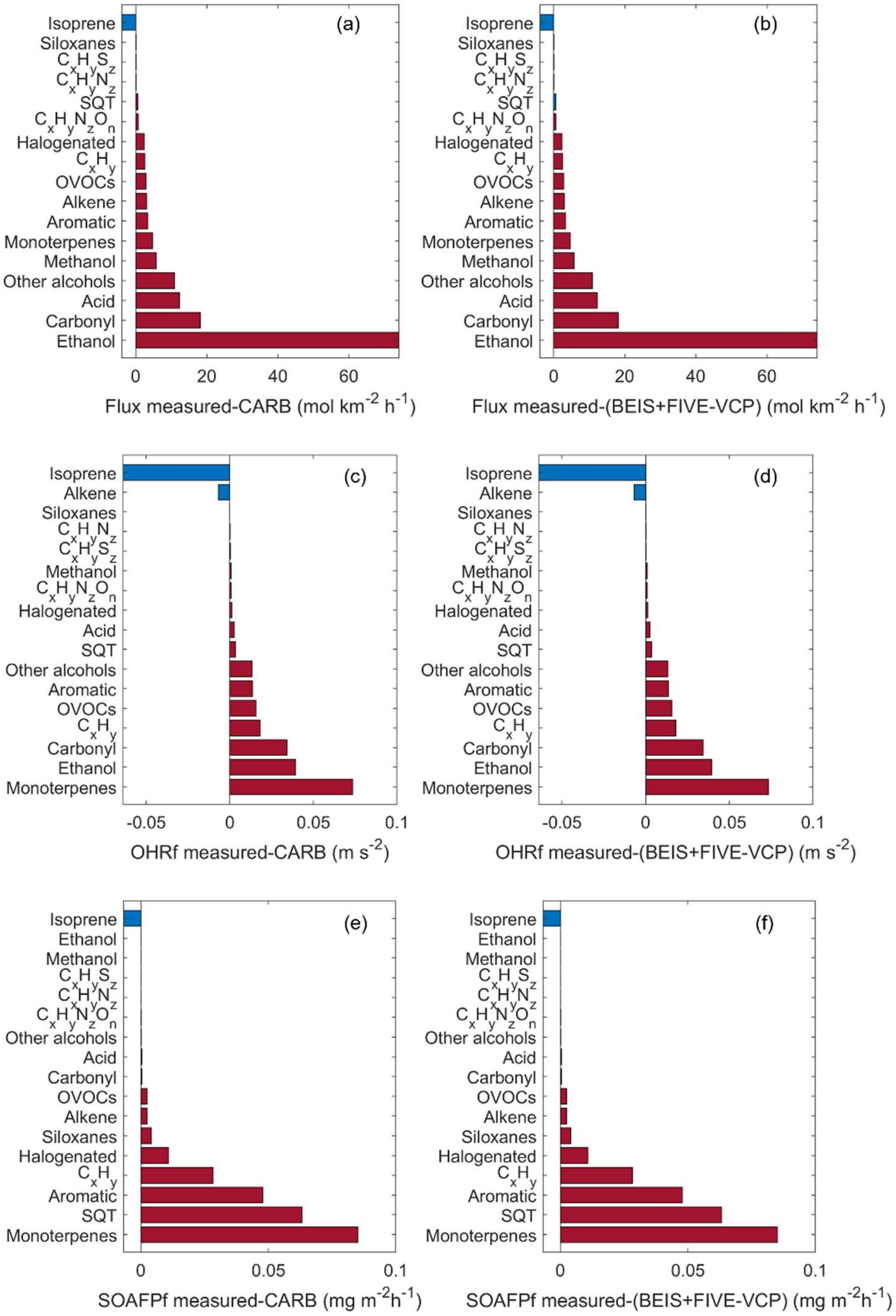

Figure S3. Relevance of the discrepancies between measurements and inventories for the total molar flux (a, b), the OH reactivity of the emissions (c, d), and the SOA formation potential of the emissions (e, f). The bars show the difference between measurements and inventories (a, c, e for CARB, and b, d, f for BEIS+ FIVE-VCP).

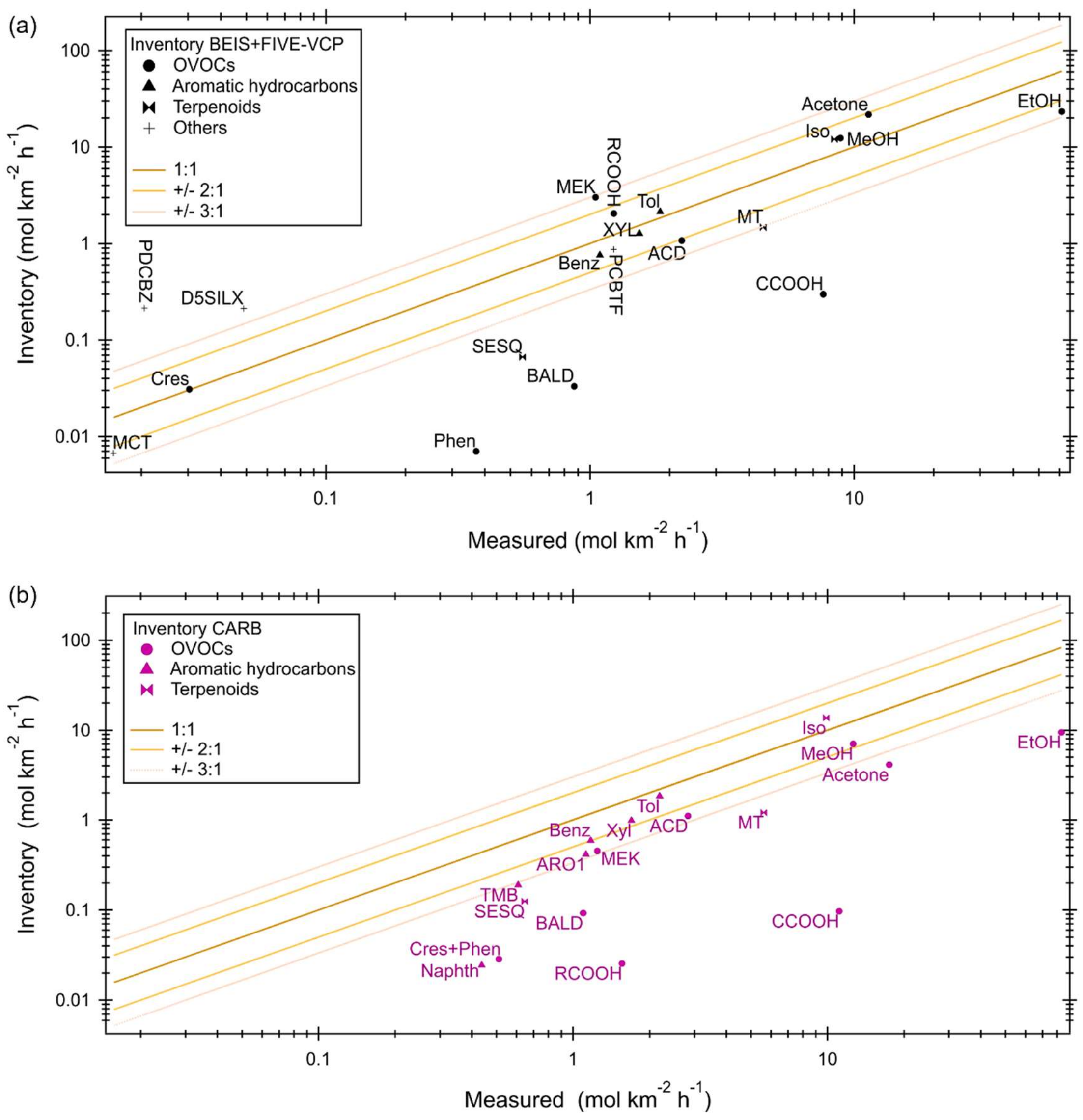

Figure S4. Comparison of mean values between measured and inventory emissions of individual VOCs for (a) BEIS+FIVE-VCP and (b) CARB. Cres: Cresol, Phen: Phenol, MCT: Methanethiol, PDCBZ: Paradichlorobenzene, D5SILX: D5 siloxane, SESQ: sesquiterpenes, TMB: trimethylbenzene, BALD: benzaldehyde, Naphth: naphathalene, MEK: methyl ethyl ketone, Benz: benzene, PCBTF: para-chlorobenzotrifluoride, Xyl: Xylene, Tol: Toluene, MT: monoterpenes, Iso: isoprene, MeOH: methanol, EtOH, ethanol, CCOOH: acetic acid, ACD: acetaldehyde, RCOOH: Higher organic acids, ARO1: Other aromatics with kOH < $2x10^4$ ppm^{-1} min^{-1}. "Measured" values can slightly differ in comparison to each inventory because of a different distribution and coverage of inventory grid cells.

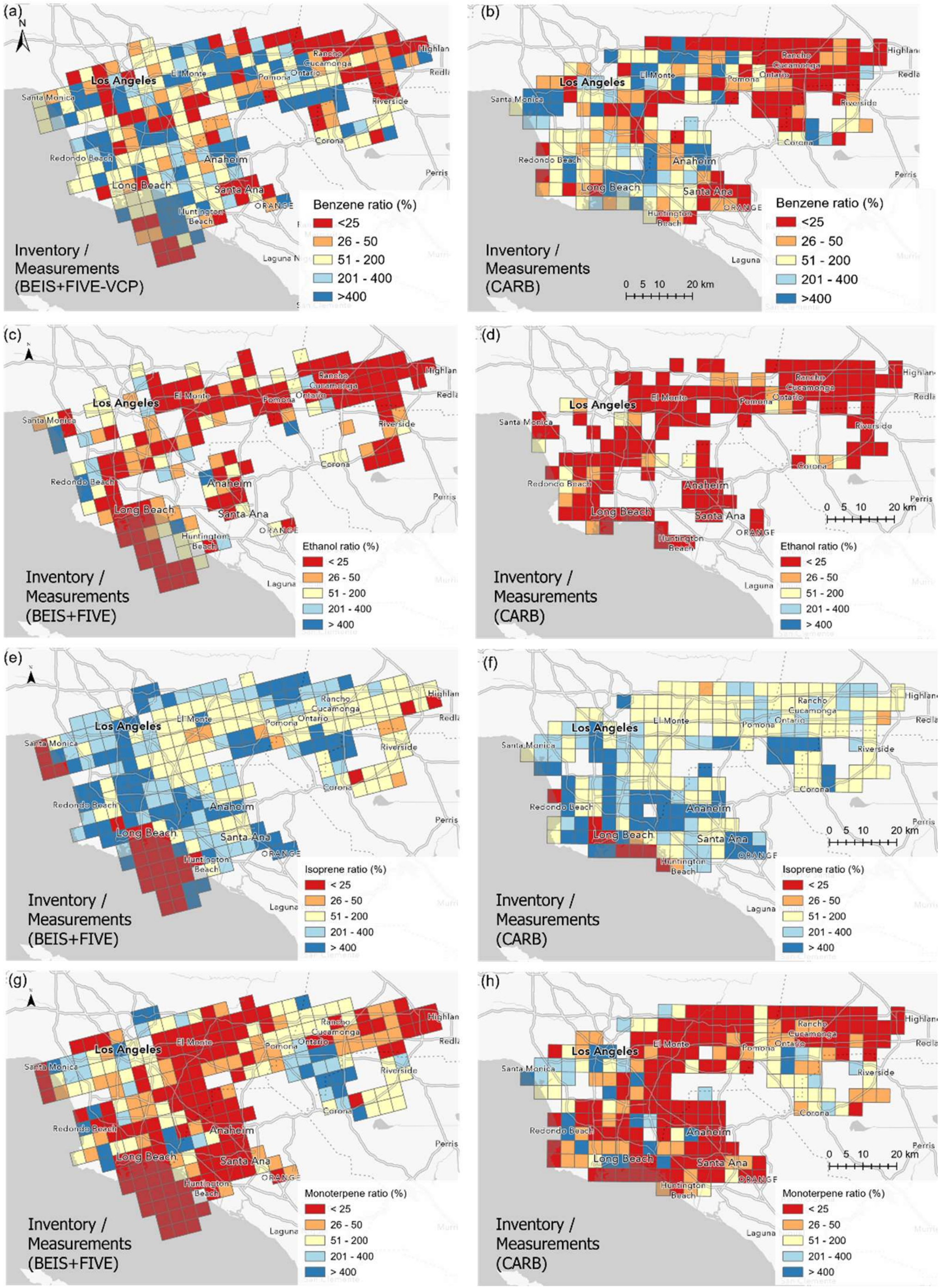

Figure S5. Ratio of inventory/measured flux in % for a) BEIS+FIVE-VCP, benzene, b) CARB, benzene, c) BEIS+FIVE-VCP, ethanol, d) CARB, ethanol, e) BEIS+FIVE-VCP, isoprene, f) CARB, isoprene, g) BEIS+FIVE-VCP, monoterpenes, h) CARB, monoterpenes. Blue colors show that the measurements were lower than the inventory, red colors that the measurements were higher than the inventory.

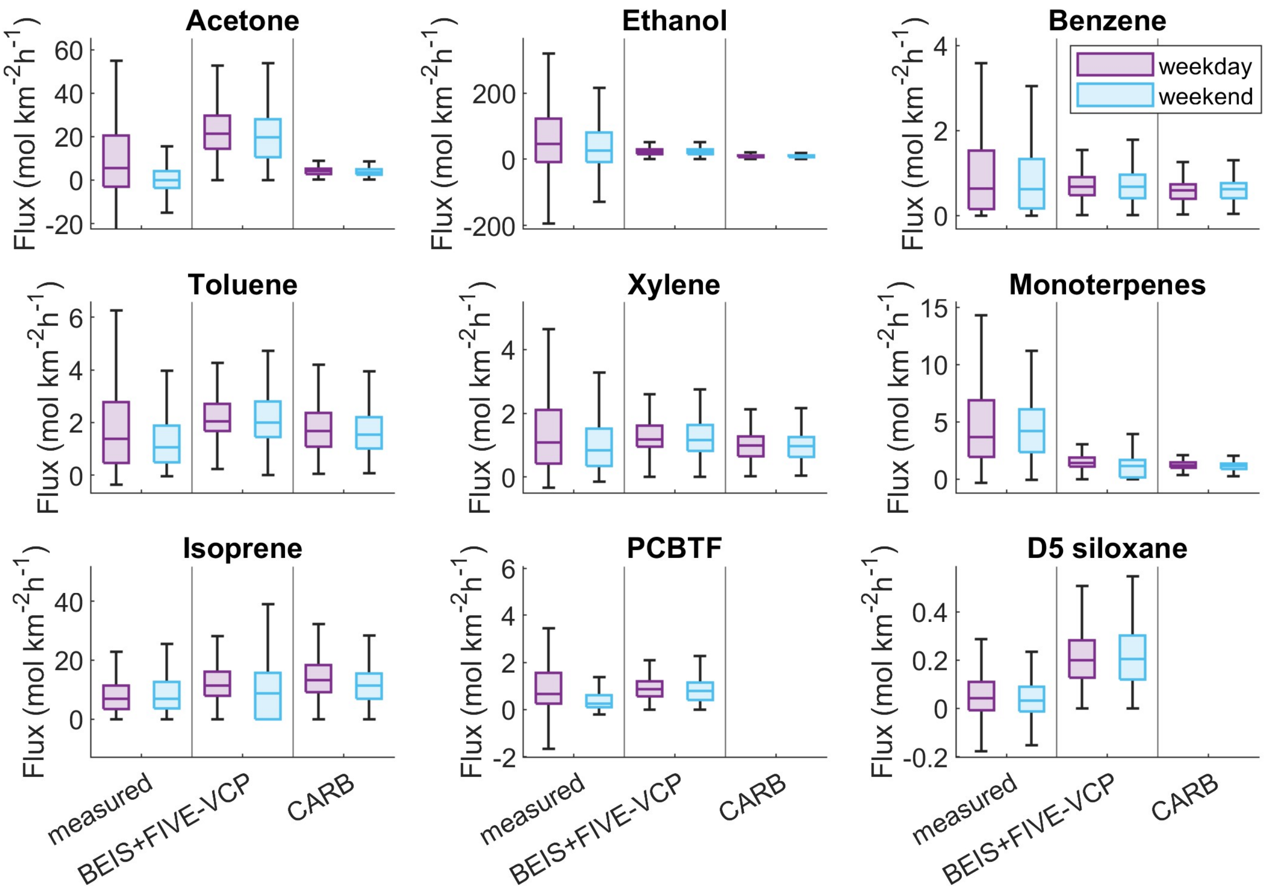

Figure S6. Weekday and weekend fluxes shown for a selection of VOCs in comparison between measurements and the two inventories. The boxes represent the 25th-75th percentile of the data, the whiskers the 5th-95th percentile, and the horizontal line the median. PCBTF and D5 siloxane are not reported in the CARB inventory. The plot only includes data from inventory grid cells that were sampled both on weekdays and weekends.

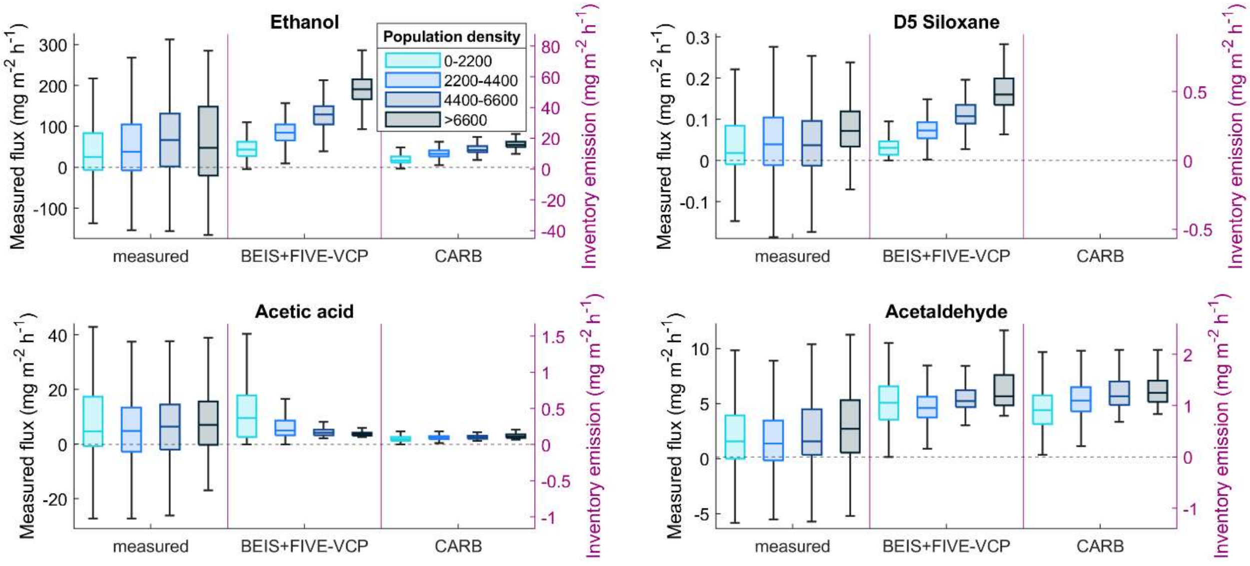

Figure S7. Fluxes of relevant indoor-to-outdoor emitted VOCs (Arata et al., in prep.) grouped as a function of population density. Population density is given in people per km². The left y axis corresponds to the measured data, the right y axis to the two inventories (note significant scale differences between the two axes).

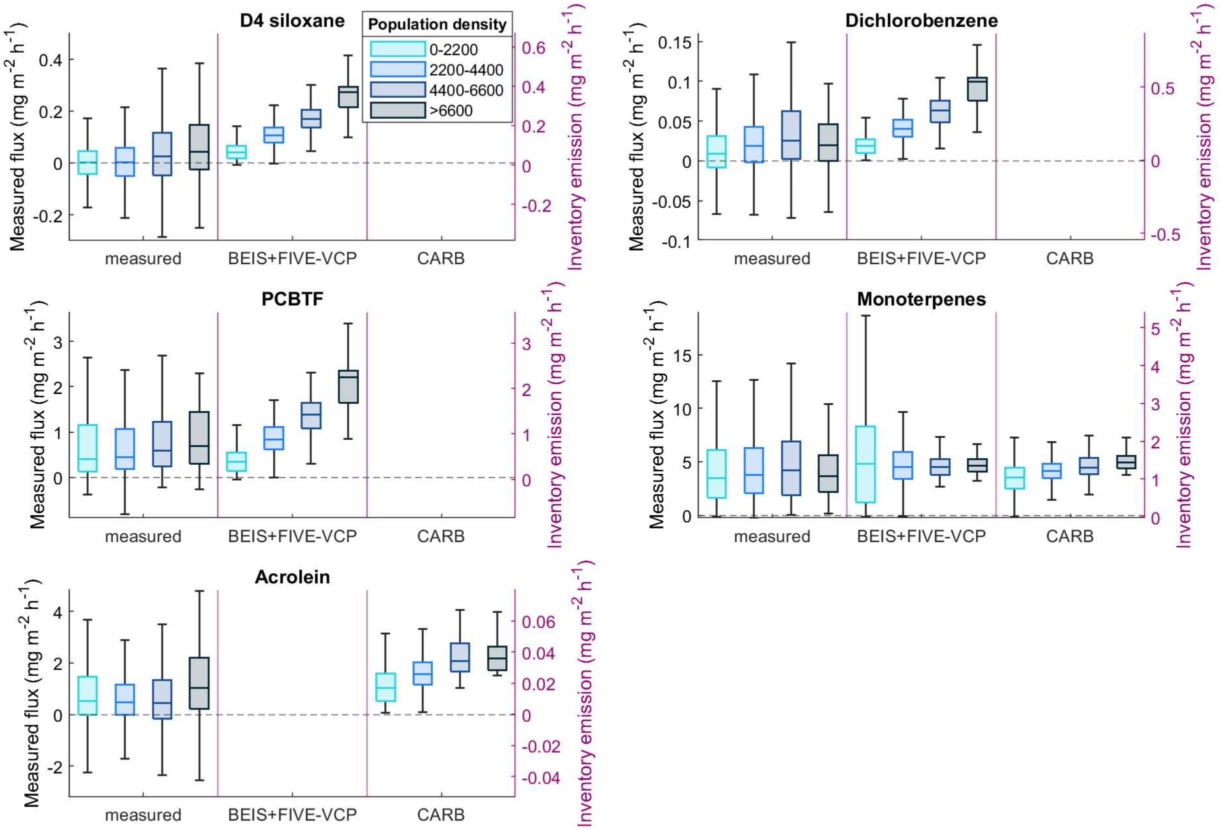

Figure S8. Fluxes of further VOCs that were reported to increase relative to population density in a previous study (*18*), and acrolein as a potential frying/cooking marker (*19*), grouped as a function of population density. Population density is given in people per km². The left y axis corresponds to the measured data, the right y axis to the two inventories (note significant scale differences between the two axes).

Supplementary Table 3. Mean to median ratio (sorted from highest to lowest) for mass fluxes of some important VOCs that are part of at least one of the inventories. RCOOH: Higher organic acids, ARO1: Other aromatics with kOH < 2×10^4 ppm^{-1} min^{-1}. VOCs with strong localized point sources are expected to have higher mean to median ratios than VOCs that are emitted consistently throughout the study area. Acetone, PCBTF and methyl ethyl ketone are probably emitted through localized solvent use, and methanethiol from wastewater treatment, while the aromatics have strong point sources over refineries, chemical manufacturing plants, and oil/gas wells.

**VOC**	**Mean to Median ratio**
**Acetone**	7.18
PCBTF	2.68
Methanethiol	1.89
Methyl ethyl ketone	1.83
D4 siloxane	1.81
Benzene	1.80
C3 benzenes	1.65
C2 benzenes	1.64
Toluene	1.58
Ethanol	1.56
Sesquiterpenes	1.54
Acetic acid	1.53
Acetaldehyde	1.51
Cresol	1.45
Isoprene	1.42
Acrolein	1.42
D5 siloxane	1.39
Dichlorobenzene	1.36
Naphthalene	1.35
Phenol	1.35
Methanol	1.33
RCOOH	1.32
Nonanal	1.29
Monoterpenes	1.26
ARO1	1.25
Benzaldehyde	1.22

Supplementary Table 4. Results of the linear regression (median flux vs. median population density) for the VOCs shown in Fig. S7 and S8 for the four population bins. The ratio of the regression slope/uncertainty shows that all listed VOC flux medians show a significant increase with population density (within the 1σ uncertainty) except the monoterpenes, whose slope is therefore shown in brackets. The slopes also provide estimates of the VOC emission per person and hour. It must be noted that the original data from which the medians are calculated have such a large scatter that those data do not show a significant increase with population density.

VOC	Slope (mg person^{-1} h^{-1})	1 σ uncertainty of slope (mg person^{-1} h^{-1})	Ratio slope/ uncertainty
Ethanol	4170.0	3120.0	1.3
D5 siloxane	7.7	1.9	4.1
Acetic acid	419.0	86.0	4.9
Acetaldehyde	186.6	88.9	2.1
D4 siloxane	6.8	1.5	4.6
Dichlorobenzene	1.6	1.3	1.2
PCBTF	47.8	6.1	7.8
Monoterpenes	(40.0)	76.0	0.5

References

1. McDonald, B. C.; McBride, Z. C.; Martin, E. W.; Harley, R. A. High-resolution mapping of motor vehicle carbon dioxide emissions. *Geophys Res Atmos* **2014,** *119* (9), 5283–5298. DOI: 10.1002/2013JD021219.

2. McDonald, B. C.; McKeen, S. A.; Cui, Y. Y.; Ahmadov, R.; Kim, S.-W.; Frost, G. J.; Pollack, I. B.; Peischl, J.; Ryerson, T. B.; Holloway, J. S.; Graus, M.; Warneke, C.; Gilman, J. B.; Gouw, J. A. de; Kaiser, J.; Keutsch, F. N.; Hanisco, T. F.; Wolfe, G. M.; Trainer, M. Modeling Ozone in the Eastern U.S. using a Fuel-Based Mobile Source Emissions Inventory. *Environ. Sci. Technol.* **2018,** *52* (13), 7360–7370. DOI: 10.1021/acs.est.8b00778.

3. Harkins, C.; McDonald, B. C.; Henze, D. K.; Wiedinmyer, C. A fuel-based method for updating mobile source emissions during the COVID-19 pandemic. *Environ. Res. Lett.* **2021,** *16* (6), 65018.

4. McDonald, B. C.; Dallmann, T. R.; Martin, E. W.; Harley, R. A. Long-term trends in nitrogen oxide emissions from motor vehicles at national, state, and air basin scales. *J. Geophys. Res.* **2012,** *117* (D21), 1-11. DOI: 10.1029/2012JD018304.

5. McDonald, B. C.; Gentner, D. R.; Goldstein, A. H.; Harley, R. A. Long-term trends in motor vehicle emissions in u.s. urban areas. *Environ. Sci. Technol.* **2013,** *47* (17), 10022–10031. DOI: 10.1021/es401034z.

6. US EPA. *2017 National Emissions Inventory*. https://www.epa.gov/air-emissions-inventories/2017-national-emissions-inventory-nei-data.

7. McDonald, B. C.; Gouw, J. A. de; Gilman, J. B.; Jathar, S. H.; Akherati, A.; Cappa, C. D.; Jimenez, J. L.; Lee-Taylor, J.; Hayes, P. L.; McKeen, S. A.; Cui, Y. Y.; Kim, S.-W.; Gentner, D. R.; Isaacman-VanWertz, G.; Goldstein, A. H.; Harley, R. A.; Frost, G. J.; Roberts, J. M.; Ryerson, T. B.; Trainer, M. Volatile chemical products emerging as largest petrochemical source of urban organic emissions. *Science (New York, N.Y.)* **2018,** *359* (6377), 760–764. DOI: 10.1126/science.aaq0524.

8. Francoeur, C. B.; McDonald, B. C.; Gilman, J. B.; Zarzana, K. J.; Dix, B.; Brown, S. S.; Gouw, J. A. de; Frost, G. J.; Li, M.; McKeen, S. A.; Peischl, J.; Pollack, I. B.; Ryerson, T. B.; Thompson, C.; Warneke, C.; Trainer, M. Quantifying Methane and Ozone Precursor Emissions from Oil and Gas Production Regions across the Contiguous US. *Environ. Sci. Technol.* **2021,** *55* (13), 9129–9139. DOI: 10.1021/acs.est.0c07352.

9. Coggon, M. M.; Gkatzelis, G. I.; McDonald, B. C.; Gilman, J. B.; Schwantes, R. H.; Abuhassan, N.; Aikin, K. C.; Arend, M. F.; Berkoff, T. A.; Brown, S. S.; Campos, T. L.; Dickerson, R. R.; Gronoff, G.; Hurley, J. F.; Isaacman-VanWertz, G.; Koss, A. R.; Li, M.; McKeen, S. A.; Moshary, F.; Peischl, J.; Pospisilova, V.; Ren, X.; Wilson, A.; Wu, Y.; Trainer, M.; Warneke, C. Volatile chemical product emissions enhance ozone and modulate urban chemistry. *PNAS* **2021,** *118* (32), 1–9. DOI: 10.1073/pnas.2026653118.

10. Langford, B.; Davison, B.; Nemitz, E.; Hewitt, C. N. Mixing ratios and eddy covariance flux measurements of volatile organic compounds from an urban canopy (Manchester, UK). *Atmos. Chem. Phys.* **2009,** *9* (6), 1971–1987. DOI: 10.5194/acp-9-1971-2009.

11. Langford, B.; Nemitz, E.; House, E.; Phillips, G. J.; d. Famulari; Davison, B.; Hopkins, J. R.; Lewis, A. C.; Hewitt, C. N. Fluxes and concentrations of volatile organic compounds above central London, UK. *Atmos. Chem. Phys.* **2010,** *10* (2), 627–645. DOI: 10.5194/acp-10-627-2010.

12. Velasco, E.; Pressley, S.; Grivicke, R.; Allwine, E.; Coons, T.; Foster, W.; Jobson, B. T.; Westberg, H.; Ramos, R.; Hernández, F.; Molina, L. T.; Lamb, B. Eddy covariance flux measurements of pollutant gases in urban Mexico City. *Atmos. Chem. Phys.* **2009,** *9* (19), 7325–7342. DOI: 10.5194/acp-9-7325-2009.

13. Park, C.; Schade, G. W.; Boedeker, I. Flux measurements of volatile organic compounds by the relaxed eddy accumulation method combined with a GC-FID system in urban Houston, Texas. *ATMOSPHERIC ENVIRONMENT* **2010,** *44* (21-22), 2605–2614. DOI: 10.1016/j.atmosenv.2010.04.016.

14. Valach, A. C.; Langford, B.; Nemitz, E.; MacKenzie, A. R.; Hewitt, C. N. Seasonal and diurnal trends in concentrations and fluxes of volatile organic compounds in central London. *Atmos. Chem. Phys.* **2015,** *15* (14), 7777–7796. DOI: 10.5194/acp-15-7777-2015.

15. Rantala, P.; Järvi, L.; Taipale, R.; Laurila, T. K.; Patokoski, J.; Kajos, M. K.; Kurppa, M.; Haapanala, S.; Siivola, E.; Petäjä, T.; Ruuskanen, T. M.; Rinne, J. Anthropogenic and biogenic influence on VOC fluxes at an urban background site in Helsinki, Finland. *Atmos. Chem. Phys.* **2016,** *16* (12), 7981–8007. DOI: 10.5194/acp-16-7981-2016.

16. Karl, T.; Striednig, M.; Graus, M.; Hammerle, A.; Wohlfahrt, G. Urban flux measurements reveal a large pool of oxygenated volatile organic compound emissions. *Proceedings of the National Academy of Sciences of the United States of America* **2018,** *115* (6), 1186–1191. DOI: 10.1073/pnas.1714715115.

17. Acton, W. J. F.; Huang, Z.; Davison, B.; Drysdale, W. S.; Fu, P.; Hollaway, M.; Langford, B.; Lee, J.; Liu, Y.; Metzger, S.; Mullinger, N.; Nemitz, E.; Reeves, C. E.; Squires, F. A.; Vaughan, A. R.; Wang, X.; Wang, Z.; Wild, O.; Zhang, Q.; Zhang, Y.; Hewitt, C. N. Surface–atmosphere fluxes of volatile organic compounds in Beijing. *Atmos. Chem. Phys.* **2020,** *20* (23), 15101–15125. DOI: 10.5194/acp-20-15101-2020.

18. Gkatzelis, G. I.; Coggon, M. M.; McDonald, B. C.; Peischl, J.; Aikin, K. C.; Gilman, J. B.; Trainer, M.; Warneke, C. Identifying Volatile Chemical Product Tracer Compounds in U.S. Cities. *Environ. Sci. Technol.* **2021,** *55* (1), 188–199. DOI: 10.1021/acs.est.0c05467.

19. Alves, C. A.; Evtyugina, M.; Cerqueira, M.; Nunes, T.; Duarte, M.; Vicente, E. Volatile organic compounds emitted by the stacks of restaurants. *Air Qual Atmos Health* **2015,** *8* (4), 401–412. DOI: 10.1007/s11869-014-0310-7.
